# Editorial: Maximal Neuromuscular Capacities: Relevance to Daily Function and Athletic Performance

**DOI:** 10.3389/fphys.2022.908611

**Published:** 2022-05-13

**Authors:** Mehmet Uygur, Selcuk Akpinar, Predrag R. Bozic, Stevo Popovic, Nejc Sarabon

**Affiliations:** ^1^ Health and Exercise Department, Rowan University, Glassboro, NJ, United States; ^2^ Physical Education and Sports Department, Nevşehir Hacı Bektaş Veli University, Nevşehir, Turkey; ^3^ Serbian Institute of Sport and Sports Medicine (SISSM), Belgrade, Serbia; ^4^ University of Montenegro, Faculty for sport and Physical Education, Niksic, Montenegro; ^5^ Western Balkan Sport Innovation Lab, Podgorica, Montenegro; ^6^ Faculty of Health Sciences, University of Primorska, Koper, Slovenia

**Keywords:** neuromuscular capacity, muscle force, clinical populations, athletes, older adults, strength, power

## Introduction

Humans generate muscle forces of different qualities to meet the requirements of the attempted tasks. While some of the athletic tasks require the generation of maximal forces or rates of force development, some other athletic and daily tasks require the generation of submaximal forces quickly. Therefore, assessment of the maximal capacities of the neuromuscular system and creating training methods to improve them is of importance in athletic and clinical populations. This special topic published seven studies conducted by 36 authors and contributed to the advancement of the research area by conducting studies in the various assessment techniques of neuromuscular capacities and providing novel training methods to improve them.

## Assessment of Neuromuscular Capacities

The level of symmetry between limbs relates to athletic performance and could be an indicator for an injury risk. While symmetry has been assessed using either maximal force or rates of force development (RFD), Smajla et al. investigated it when individuals generate submaximal forces pulses. The studied variables included RFD scaling factor (RFD-SF) and rate of force relaxation scaling factor (RFR-SF). In this large-scale study, there were 248 male and female participants. The results suggested higher RFD-SF and lower symmetry values for RFR-SF in males than females, and higher symmetry values for RFR-SF in tennis players compared to those values in basketball players and non-athletes.

Having standards and norms are important when it comes to comparisons of strength values across different populations or to evaluate the outcomes of rehabilitative, therapeutic interventions and strength and conditioning. One of the commonly tested muscle groups is the knee extensors and flexors since they contribute to the performance of most of the daily and athletic tasks and there is a need to develop reference values for the maximal values for those muscle groups. In the meta-analysis by Sarabon et al. authors created reference values for the relative strength in younger and older adults of both sexes using the data from 411 studies and 13,893 participants. Results revealed that relative strength was higher in adult males than adult females and that the younger adults were stronger than the older adults even when their strength values were normalized to their body mass.

Change of direction (CoD) in athletic tasks requires quick and accurate movements and contributes to athletic performance. During CoD maneuvers, athletes are required to generate quick acceleration and deceleration, both of which could only be accurately measured by sophisticated tools such as motion capture systems. Therefore, the development of new technologies that are feasible and accurate in CoD testing is very important. Eriksrud et al. investigated the validity of a motorized resistance device (MRD) to measure the velocity of individuals during CoD by comparing them to the data obtained from a motion capture system. Results revealed excellent correlation values between MRD and motion capture measurements along with low biases and coefficient of variation values, suggesting MRD as a valid tool to measure velocity during CoD.

## Improving Neuromuscular Capacities

Humans engage in different resistance training activities to increase the maximal capacities in strength or power production. In this type of training, there could be specific effects of weight and inertia of the lifted object. Duric et al. investigated the improvements in maximal strength and power after the completion of 8-weeks bench-press throw (BPT) training using constant resistance, inertial resistance, and their combination. Results in general suggested that the gains in power after the completion of BPT training could be specific to the training method used, while the gains in strength could be similar across the training protocols. Authors concluded that when the goal is to improve the performance of rapid movements, the preferred method should be based on the inertia-based resistance types. When the goal is to maximize strength, constant resistance training should be used.

It is well-known that older adults benefit from engaging in aerobic exercise. Participating in such a training could have a variety of benefits that include improvements in cardiovascular and neuromuscular systems and the performance in the activities of daily living. In the study of Bai et al., authors conducted a systematic review on the effects of combining strength/resistance training with aerobic training on the physical performance of older adults. Analyzing 18 articles that met their inclusion criteria, authors concluded that the aerobic training that is combined with strength/resistance training have more benefits in overall physical function, including walking speed, lower extremity strength, and body fat than those benefits obtained from participating only in resistance/strength training or in aerobic training. Therefore, authors concluded that older adults should engage in training that includes multiple components of physical fitness.

Functional training is an important way to improve physical fitness in the athletic population. In the systematic review of Xiao et al., the authors investigated the effects of functional training on various properties of physical fitness in athletic populations. This review paper analyzed nine papers that met the inclusion criteria and concluded that there is strong evidence for the benefits of functional training in improving strength, power, balance, and agility and moderate evidence for improving flexibility and muscular endurance.

Resistance training (RT) performed with eccentric muscle contractions are known to be an effective strategy to induce neuromuscular adaptations. However, this type of training is associated with muscle soreness and risk of injury, which limits the use of eccentric RT in older adults. In their perspective article, Harper and Thompson discussed the potential use of minimal dose eccentric RT to alleviate sarcopenia and age-related decrease in muscle and physical function in older adults. Authors concluded that minimal dose eccentric RT could increase adherence of older adults in RT and serve as an effective strategy to improve neuromuscular and physical functioning in older adults.

## Conclusion

Current Research Topic contributes to existing literature by providing information on between limb symmetries in quick submaximal force production and standard values for knee strength for various populations. It also revealed the importance of functional training in athletic populations and resistance training combined with aerobic training and minimum load eccentric training in older adults. Finally, it provided the validity of a simple device to measure change of direction in athletic populations. The published papers of this Research Topic raised important questions for the future studies that include 1) exploring the external validity and sensitivity of the isometric tests in the assessment of neuromuscular functioning as well as inter-limb symmetry in various athletic and clinical populations. 2) exploring the impact of different combination of exercises (e.g., balance and endurance) in the elderly and individuals with different types of diseases, 3) exploring the effectiveness of various training principles of eccentric training (e.g., intensity, frequency) in the improvement of the neuromuscular capacities in the elderly, 4) examining the effects of functional training on the components of physical fitness among different types of athletes, and 5) investigating the roles of antagonist muscle during training programs performed with constant, inertial, and combined loads.

